# Drugs and alcohol Use patterns among those seeking care in urban rehabilitation centres before and during early months of COVID-19 in Uganda

**DOI:** 10.4314/ahs.v22i2.15S

**Published:** 2022-08

**Authors:** Nazarius Mbona Tumwesigye, Ponsiano Ocama, David Basangwa, Flavia Matovu, Catherine Abbo, Twaibu Wamala, Claire Biribawa, Cissie Namanda, Joshua Blessing, Ronald Twesigomwe

**Affiliations:** 1 Makerere University School of Public Health, Department of Epidemiology and Biostatistics; 2 Makerere University School of Medicine, Department of Internal Medicine; 3 Butabika National Referral Mental Hospital; 4 Makerere University School of Medicine, Department of Psychiatry; 5 Uganda Harm Reduction Network

**Keywords:** Covid 19, substance use, rehabilitation centres, drugs, alcohol, interrupted time series, modified Poisson regression

## Abstract

**Introduction:**

There is a rise in alcohol and other drug (AOD) abuse in the country but details of the practice are scanty. This paper provides characteristics of clients in the rehabilitation centres, their AOD related practices before and early months of COVID-19, and correlates of repeat treatment.

**Methods:**

The study was conducted in 10 rehabilitation centres in Kampala Metropolitan area. Characterization of AOD clients involved descriptive analysis while comparison of AOD related practices pre-and during COVID-19 lockdown was carried out using interrupted time series analysis. Modified Poisson regression model was used to analyse the repeat treatment.

**Results:**

The clients were mostly male (85%), single (57%) and had attained secondary education (84%). Nearly a third of them (29%) were unemployed while 68% were aged between 15–34 years. The commonest substances used were alcohol (52%), cannabis (19%), cocaine (13%) and opioids (8%). The commonest sources of substances were street dealers (52%) and friends (37%). COVID-19 did not change the pattern of AOD use except for Opioids. Repeat treatment was associated with being male, seeking care in private facilities, being casual labourer/self-employed.

**Conclusion:**

Intervention programs should target the educated, the unemployed, young men, their friends, street drug dealers and AOD hotspots.

## Introduction

The global burden of disease caused by harmful use of alcohol and other drugs (AOD) is enormous. AOD abuse is one of the leading risk factors for detrimental population health worldwide^1^. Alcohol and other drug abuse are among the major contributors to the burden of disease in over 200 health conditions. This exceeds those caused by many other risk factors and diseases high on the global health agenda^2^. This ultimately has a direct impact on many health-related targets of the Sustainable Development Goals (SDGs)

AOD abuse is linked to many non-communicable diseases like cardiovascular diseases, to road traffic injuries, violence and suicide^3^, to diseases associated with risky sexual behaviours^1^, and poor mental health^4^. Globally this makes AOD abuse to be responsible for 7.2% of all premature (among persons 69 years of age and younger) mortality in 2016. People of younger ages were disproportionately affected by alcohol compared to older persons, and 13.5% of all deaths among those aged 20–39 years of age is attributed to alcohol^2^.

Although the highest levels of alcohol consumption are in Europe, Africa bears the heaviest burden of disease and injury attributed to alcohol. Alcohol per capita consumption per year in litres of pure alcohol is one of two indicators for SDG health target 3.5^5^. According to the 2018 Global status report on Alcohol and health, the total Alcohol per capita for Uganda in the year 2016 was 9.5 compared to 6.3 for the WHO Africa region^6^.

Half of the admissions in the Ugandan National Mental Referral Hospital are young people with AOD use disorders^7^. AOD is on the rise in Uganda especially among young people^4^. Alcohol use is among the top 10 risk factors that contribute to the country's burden of disease^8^. This observation should compel major interest in the prevention of AOD abuse in Uganda but it does not get the attention it deserves. A review of literature shows there is evidence gap in relation to patterns, differentials and trends in AOD use. While there is some evidence on alcohol use information on other drugs is grossly lacking. In this work we characterise clients who reported at 10 rehabilitation centres in Kampala metropolitan area in a period of 8 months and assess patterns, differentials and trends of AOD practices covering 8 months of observation at the centres. In addition, the paper compares the levels of AOD practices before and during early months of COVID-19 lockdown measures and established factors associated with repeat treatment for AOD.

The study coincided with the onset of COVID-19 epidemic in Uganda. The first case was reported on the 21^st^ March 2020 and as of 30^th^ June 2020 there were 1074 confirmed cases with no deaths as yet^9^. Stringent measures against the pandemic were taken in March and from 1st of April there was a total nationwide lock down that was eased in June 2022. All forms of public transport were suspended during lockdown except for cargo planes, trucks and trains and restrictions on private vehicle movements were also instituted^10^.

## Materials and methods

### Study site and population

Ten facilities located in Kampala Metropolitan Area (KMA) involved in the treatment of alcohol and drug abuse were selected according to the following criteria: having established accommodation for clients and having record keeping facilities including a computer, storage for records and a staff to enter data. An internet search for rehabilitation centres in Kampala metropolitan area that includes the city and the surrounding district of Wakiso, followed by phone calls led to the following facilities: Africa Retreat Centre, Butabika National Referral Hospital, Fore Tranquil Homes, Hope and Beyond, Life Back Foundation, Recovery Solutions, Safe Places, Serenity Centre, National Care Centre and Uganda Harm Reduction Network. The study units were all clients of the rehabilitation centres.

KMA has a population estimate of about 4,385,900 (Wakiso district, 2,735,100 and Kampala, 1,650,800)^11^. The area is served by several AOD related rehabilitation centres but most of them are small and not yet fully established.

### Sample size and sampling procedure

All clients of the 10 facilities in Kampala Metropolitan area seeking AOD treatment services in period of November 2019 to June 2020 were included in the study.

### Study variables

The variables of interest included, type of substance used, background characteristics of the clients, source of referral, mode of use, testing for HIV and hepatitis C, rural/urban residence, source of payment for treatment bills and repeat treatment.

### Preparation for Data collection

Clinicians at the facilities were trained on how to identify persons with hazardous and harmful patterns of alcohol and drug consumption using the AUDIT tool (Alcohol Use Disorders Identification Test)^12^. The audit tool and a few more questions were added on the client screening form. Record Clerks were trained on how to abstract data from client files and enter it into a Microsoft access designed database.

### Data Collection Tools

A standardized paper-based data collection tool to capture key components on the variables of interest extracted from patient files was developed and used. A database with in-built range and consistency checks was designed in Mcrosoft Access and installed on computers at each of the ten participating facilities. The data base had inbuilt range and consistency checks.

### Data management and analysis

The data were exported from Microsoft Access database to Excel from where they were exported to Stata Version 14 for cleaning and analysis. To characterize the clients to the rehabilitation facilities charts and cross-tabulations were used to show different patterns and differentials of the clients as well as trend for the 8 months. Key among variables examined weretype and source of drugs, and frequency of consumption, use of injecting drugs, reasons for starting and sustaining drug use.

To get a statistical difference between before and after intervention (instituting of lockdown) an interrupted time series analysis was applied. It's a kind of analysis that compares the level and trend of the data before and after intervention. The time series refers to the data over the period, while the interruption is the intervention, which is a controlled external influence or set of influences^13^. Changes in level and trend are expected in a period subsequent to introduction of the intervention^14^. Interrupted time series (ITS) analysis is a strong quasi experimental design that can be used to evaluate the effectiveness of a population-level intervention that is clearly defined at a given time point^15^.

## Ethical considerations

The study team obtained approval from Makerere University School of Public Health Higher Degrees, Research and Ethics Committee as well National Council of Science and Technology. Administrative clearance was also obtained from the participating facilities. The management team of each of the facilities was informed that their facilities could stop participating in the research at any time if they so wished. They were also given an opportunity to ask questions related to the study or the facility's rights as a participant. All data were de-identified from facilities to protect confidentiality of the clients/patients.

## Results

### Background characteristics of study clients

The overall average age of clients in all the ten facilities was 32 years with 45% of the clients in the 25–34 age group and 23% under 25 years. Most of the clients were men (85%), had attained at least secondary level of education (84%) while those with no education were only 5.6%. More than half of the clients were single (57%), nearly a third (29%) were unemployed while majority were Christians (protestants, 45% and Catholics, 35%) and Urban residents (87%) (Table 1).

**Table 1:** Background characteristics of the clients

Characteristics	Males n (%)	Females n (%)	All n (%)
**Age**			
15–24	148(23.0)	29(24.4)	177(23.2)
25–34	291(45.2)	53(44.5)	344(45.1)
35+	205(31.8)	37(31.1)	242(31.7)
**Education**			
None	39(6.5)	1(0.9)	40(5.6)
Primary	66(11.0)	11(9.7)	77(10.8)
Secondary	321(53.7)	71(62.3)	392(55.1)
Tertiary	172(28.8)	31(27.2)	203(28.5)
**Marital status**			
Single	261(58.9)	38(48.1)	299(57.3)
Married/Living together	109(24.6)	24(30.4)	133(25.5)
Other	73(16.5)	17(21.5)	90(17.2)
**Religion**			
Catholic	220(35.2)	36(30.8)	256(34.5)
Protestant	276(44.2)	56(47.9)	332(44.7)
Muslim	84(13.4)	17(14.5)	101(13.6)
SDA	13(2.1)	2(1.7)	15(2.0)
Other	32(5.1)	6(5.1)	38(5.1)
**Employment**			
Formal/Regular	88(19.6)	10(12.5)	98(18.5)
Pupil/Student	55(12.3)	8(10.0)	63(11.9)
Unemployed	124(27.6)	27(33.8)	151(28.5)
Housewife	0(0.0)	7(8.8)	7(1.3)
Casual labour	71(15.8)	10(12.5)	81(15.3)
Self Employed	86(19.2)	16(20.0)	102(19.3)
Other	25(5.6)	2(2.5)	27(5.1)
**Area of residence**			
Urban	545(86.1)	108(90.8)	653(86.8)
Rural	88(14.0)	11(9.2)	99(13.2)
All	644(100.0)	119(100.0)	754(100.0)

Average age(sd): Male=31.9(9.7); Female =31.9; All =31.9(10.8)

### Number of Clients at facilities by months

Overall, between November 2019 and June 2020 763 clients were managed for AOD in the facilities. More than a half of the clients/patients were from Butabika National Referral Hospital (BNRMH) (51.5%) while the least number of clients registered in the same period were from Recovery solutions (2.1%) as well as Safe Places (2.2%) (Table 2). These numbers varied in each of the facilities although they remained high in BNRMH. High numbers of clients were registered in the months of February 2020 for Butabika (61%) and least in December 2019 (40%). Another facility that recorded slightly high numbers was Uganda Harm Reduction Network (UHRN) with its highest recorded numbers being in December 2019 (28.4%) followed by June (27.9%) and least in February and April 2020(12.7% and 13.1%) respectively. Other facilities recorded smaller numbers of Clients with some registering zero Clients in some months especially April 2020. For example, Africa Retreat Centre, Fore Tranquil Homes, Recovery Solutions while Safe Places registered zero Clients in May 2020. Overall, a high number of Clients was registered in November 2019 (119) followed by February 2020 (118) while the least number was recoded in April 2020 (61).

**Table 2:** Number of clients by month by facility

Treatment Centre	2019m11	2019m12	2020m1	2020m2	2020m3	2020m4	2020m5	2020m6
Butabika Nat. Ref Menta.Hosp	63(52.9)	27(40.3)	55(50.0)	72(61.0)	39(45.9)	35(57.4)	55(56.1)	45(45.2)
Serenity Centre	11(9.2)	10(14.9)	12(10.9)	9(7.6)	7(8.1)	1(1.6)	2(2.0)	3(2.9)
Africa Retreat Centre	5(4.2)	2(3.0)	10(9.1)	6(5.1)	4(4.7)	0(0.0)	2(2.0)	5(4.8)
Fore Tranquil Homes	4(3.4)	1(1.5)	2(1.8)	4(3.4)	3(3.5)	0(0.0)	2(2.0)	2(1.9)
Hope and Beyond	9(7.6)	2(3.0)	1(0.9)	4(3.4)	5(5.8)	7(11.5)	8(8.2)	5(4.8)
Life Back Foundation	1(0.8)	1(1.5)	1(0.9)	5(4.2)	1(1.2)	4(6.6)	4(4.1)	4(3.9)
National Care Centre	3(2.5)	1(1.5)	4(3.6)	0(0.0)	3(3.5)	4(6.6)	2(2.0)	2(1.9)
Recovery Solutions	3(2.5)	1(1.5)	2(1.8)	2(1.7)	4(4.7)	1(1.6)	1(1.0)	2(1.9)
Safe Places	2(1.7)	3(4.5)	4(3.6)	1(0.9)	1(1.2)	1(1.6)	0(0.0)	5(4.8)
Uganda Harm Red. Network.	18(15.1)	19(28.4)	19(17.3)	15(12.7)	19(22.1)	8(13.1)	22(22.5)	29(27.9)
Total	119(100.0)	67(100.0)	110(100.0)	118(100.0)	86(100.0)	61(100.0)	98(100.0)	104(100.0)

### Primary substance used by month

**Figure 1:**
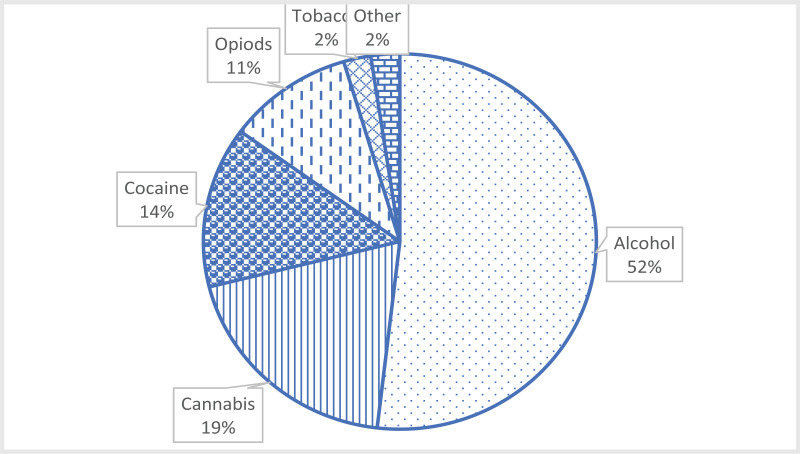
Primary drug used by clients of Kampala metropolitan rehab centres

**Figure 2:**
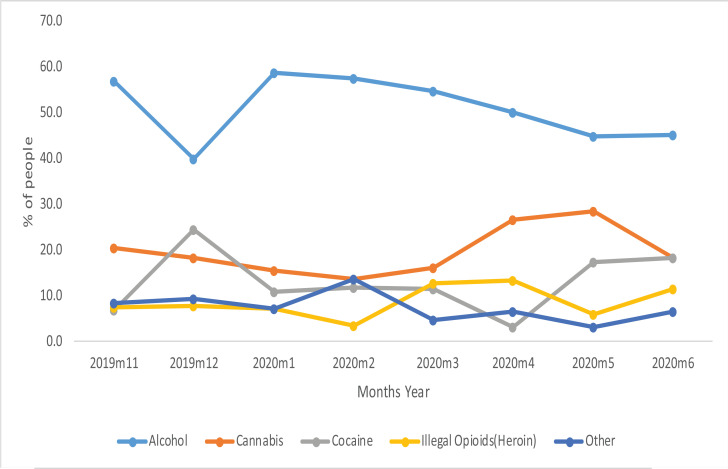
Clients that visited the facilities by month and by the primary drug used

In all the 8 months the primary substance most commonly used by the new clients to the rehabilitation centres was alcohol (52%) followed by cannabis (19%) and cocaine (14%) (Figure 1). In each of the months, alcohol use prevalence remained high among the new clients with higher percentages recorded in the months of January and February 2020 (58.7% and 57.6%) and November 2019 (56.8%). From Jan to June 2021 there was a gradual decline in proportion taking alcohol as a primary substance. For cannabis, the highest percentage was registered in the month of May 2020 (28.6%) and the least in February 2020 (13.6%). For cocaine the highest percentage was in December 2019 (24.6%) and least in April 2020 (3.3%) while for Illegal Opioids (Heroin), the highest and lowest percentages were registered in the months of April (13.3%) and February 2020 (3.4%) respectively. Beside alcohol, none of the other substances was dominant throughout the 8 months (figure 2).

**Figure 3:**
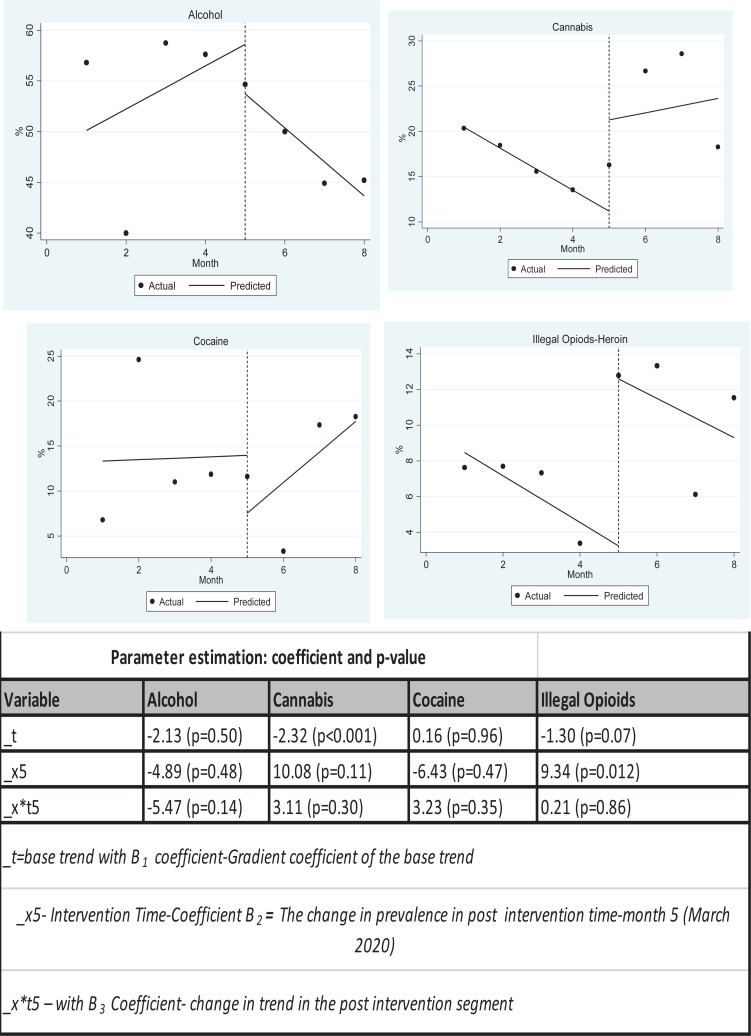
Interrupted time series analysis for % of clients that sought care at rehabilitation centres by different primary drug used with March 2020 as intervention time

An interrupted time series analysis of the trends taking March 2020 as intervention month showed no significant change in prevalence between the months April–June 2022 vs November 2021–March 2022 for Alcohol (p=0.48) Cannabis (p=0.11) and Cocaine (p=0.47). the post March 2022 prevalence for illegal opioids was on average significantly higher than that before march 2022 (p=0.012) (Figure 3).

### Primary Substance use by background characteristics

The study shows that 47% of the clients aged 35 years and more mentioned alcohol as their primary substance used compared to 40% and 13% among 25–34 years and 15–24 years respectively. The study further revealed that those whose primary substance used was alcohol were mostly men (89%), urban residents (81%), Catholic/protestants (86%) and had attained secondary education (85%) (Table 3).

**Table 3:** Use of AOD by background characteristics

Characteristic	Primary drugs Commonly used					

	Alcohol	Cannabis	Cocaine	Illegal opioids (Heroin)	Other	Total
**Age group**						
15–24	50(12.7)	62(42.5)	32(32.7)	11(17.5)	21(36.2)	176(23.2)
25–34	157(40.0)	73(50.0)	49(50.0)	36(57.1)	26(44.8)	341(45.0)
35+	186(47.3)	11(7.5)	17(17.4)	16(25.4)	11(19.0)	241(31.8)
**Sex**						
Male	350(89.1)	143(97.9)	58(59.2)	44(69.4)	45(77.6)	640(84.4)
Female	43(10.9)	3(2.1)	40(40.8)	19(30.2)	13(22.4)	118(15.6)
**Residence**						
Urban	314(81.3)	124(87.3)	98(100.0)	62(98.4)	51(87.9)	649(86.9)
Rural	72(18.7)	18(12.7)	0(0.0)	1(1.6)	7(12.1)	98(13.1)
**Religion**						
Catholic	146(38.5)	40(28.4)	27(27.6)	25(39.7)	16(28.6)	254(34.5)
Anglican/Protestant	178(47.0)	53(54.1)	53(54.1)	26(41.3)	20(35.7)	330(44.8)
Muslim	31(8.2)	28(19.9)	16(16.3)	11(17.5)	15(26.8)	101(13.7)
SDA	9(2.4)	3(2.1)	1(1.0)	1(1.6)	1(1.8)	15(2.0)
Other	15(4.0)	17(12.1)	1(1.0)	0(0.0)	4(7.1)	37(5.0)
**Education**						
None	24(6.7)	8(6.2)	1(1.0)	4(6.4)	3(5.2)	40(5.7)
Primary	32(8.9)	16(12.4)	13(13.3)	8(12.7)	8(13.8)	77(10.9)
Secondary	158(43.9)	80(62.0)	83(84.7)	44(69.8)	26(44.8)	391(55.2)
Tertiary	146(40.7)	25(19.4)	1(1.0)	7(11.1)	21(36.2)	200(28.3)

Those who took cannabis as their primary substance were younger than those who took alcohol with 50% in age group 25–34. Like those that took alcohol, these that took cannabis as their primary substance were mostly men (98%), urban (87%), protestant/catholic (83%) and had attained secondary education (81%). Similar characteristics are evident among those that took cocaine and illegal opioids but this time the proportion of women that took drugs is much higher (41% for cannabis and 30% for opioids).

The commonest source of primary drugs across all the months was street dealers (52%) followed by friends (37%), other sources (8%) and lastly from pharmacy (3%). The source of primary drugs varied in each of the months although it consistently remained high for street dealers and friends compared to other categories (Table 4). There was no observed significant change in source of primary drugs over the months.

**Table 4:** Source of primary drugs

Source	Months Year								
	
	2019 m11	2019 m12	2020 m1	2020 m2	2020 m3	2020 m4	2020 m5	2020 m6	Total (%)
Friends	35(31.0)	18(29.5)	37(35.6)	45(39.5)	31(38.3)	25(43.9)	41(44.6)	36(36.7)	268(37.2)
Street dealers	69(61.1)	37(60.7)	50(48.1)	55(48.3)	38(46.9)	22(38.6)	48(52.2)	54(55.1)	373(51.8)
Prescription /pharmacy	2(1.8)	3(4.9)	2(1.9)	6(5.3)	2(2.5)	2(3.5)	1(1.1)	2(2.0)	20(2.8)
Other	7(6.2)	3(4.9)	15(14.4)	8(7.0)	10(12.4)	8(14.0)	2(2.2)	6(6.1)	59(8.2)
Total	113(100.0)	61(100.0)	104(100.0)	114(100.0)	81(100.0)	57(100.0)	92(100.0)	98(100.0)	720(100.0)

Overall, majority of the clients (91%) used alcohol or other drugs on a daily basis. Daily substance use was most common among users of cocaine (98%) followed by alcohol (92%) (Table 5)

**Table 5:** Frequency of substance of use

Primary drug	Frequency of use		
		
	Daily	Other	Total
Cocaine	60(98.4)	1(1.6)	61(100.0)
Alcohol	244(92.4)	20(7.6)	264(100.0)
Cannabis	72(86.8)	11(13.3)	83(100.0)
Illegal opioids (Heroin)	40(88.9)	5(11.1)	45(100.0)
Other	38(86.4)	6(13.6)	44(100.0)
Total	454(91.4)	43(8.7)	497(100.0)

### Age at first use of primary drugs

**Figure 4:**
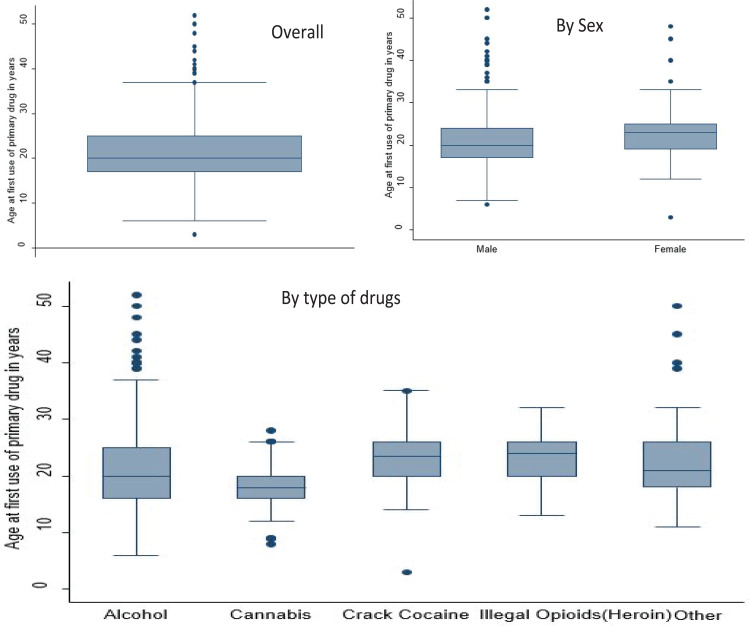
Age at first use of drugs

Figure 4 shows that the clients' median age at first use of the primary drug was about 20 years. It significandy varied by sex (p<0.001) and type of primary substance (p<0.001). Men had a lower age at first use of drugs compared to women (median: 20 vs 23) while cannabis was used much earlier (m an the rest of the drugs (median=18 years).

### Reasons for starting and sustaining substance use

**Figure 5:**
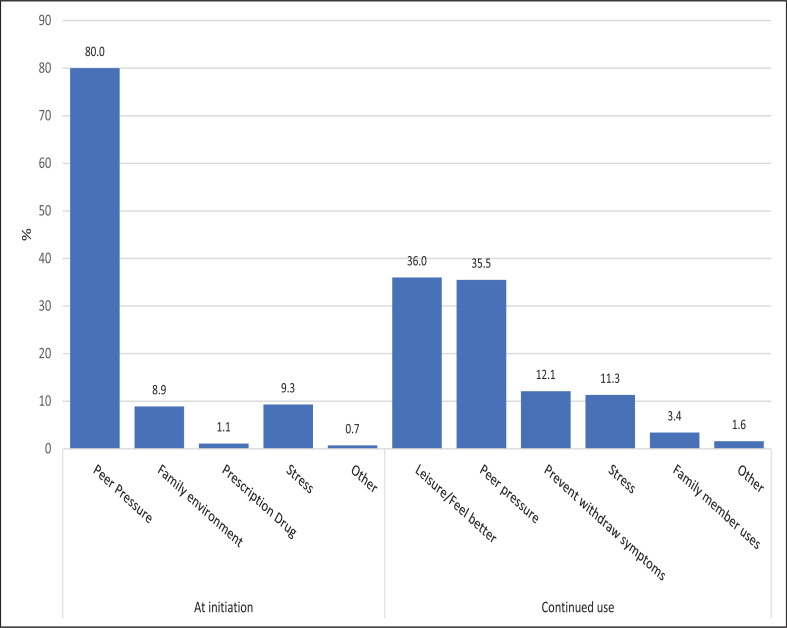
Reasons for initiating and sustaining substance use

While most of the clients (80%) started substance use out of peer pressure other factors have played a role in sustaining the habit. These include a need for leisure/feel good effect (36%), prevention of withdrawal symptoms (12%), stress (11%) and substance use by members of family (3.4%) (Figure 5).

### Injecting drug users-prevalence and sharing of equipment

**Figure 6:**
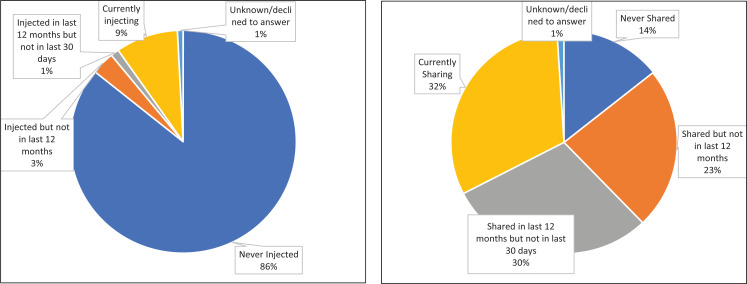
Injecting Drug users and sharing of equipment

Only 9% of the clients were injecting drug users while 3% had used injecting drugs but not in last 12 months (Figure 6). The client's average age at first injection of drugs was 25.6 years. Nearly a third (32%) of clients who had injected drugs were sharing the equipment at the time of the study (in the last 30 days) while 30% shared the equipment in last 12 months but not in previous 30 days.

The study found out that 38% of the clients had previously received treatment for alcohol/drug abuse/dependence. Of those that had been in treatment before 5% had been there for 5 or more times while 17% had been there for 3–4 times. The clients received different kinds of treatment including medical (57%), psychosocial treatment (38%), prayer (33%), traditional treatment (31%) and other kinds of treatment (3%).

Almost all facilities (9/10) had tested their clients for HIV in previous 12 months. Most of the clients (71%) had benefited from the HIV/AIDS testing services in the previous 12 months while 14% tested earlier. Conversely, hepatitis C testing services were not easily accessible as only 13% had tested in previous 12 months.

A multivariable analysis of repeat visit to the rehabilitation facilities shows that repeat visits were strongly associated with being a man, being engaged in casual labour (APR=1.63 95%CI: 1.06–2.50) or self-employed (APR=1.60, 95%CI: 1.08–2.38), having opioids as a primary drug (APR= 1.43, 95%CI: 1.04–1.96) and seeking care in private facilities(APR=1.38, 95%CI: 1.05–1.82) (Table 6).

**Table 6:** A modified Poisson Multivariable analysis for repeat visit to the rehabilitation centres

Variable	Repeat visit		Crude PR	Adj. PR
	Yes	No		
**Sex**				
Male	238(38.2)	386 (61.8)	1.0	1.0
Female	44 (37.6)	73 (62.4)	0.98 (0.76–1.27)	0.70 (0.51–0.96)*
**Age group**				
15–24	51 (29.3)	123 (70.7)	1.0	-
25–34	135 (40.2)	201 (59.8)	1.37 (1.05–1.79)*	-
35–49	77 (42.8)	103 (57.2)	1.46 (1.10–1.94)*	-
50+	20 (39.2)	31 (57.2)	1.34 (0.89–2.02)	-
**Employment**				
Regular employment	25 (26.6)	69 (73.4)	1.0	1.0
Students	14 (22.2)	49 (77.8)	0.84 (0.47–1.48)	0.78 (0.44–1.39)
Unemployed	55 (37.7)	91 (62.3)	1.42 (0.95–2.10)*	1.33 (0.90–1.97)
Casual Labour	34 (44.7)	42 (55.3)	1.68 (1.11–2.56)*	1.63 (1.06–2.50)*
Self employed	49 (49.5)	50 (50.5)	1.86 (1.26–2.75)**	1.60 (1.08–2.38)*
Other	10 (30.3)	23 (69.7)	1.14 (0.61–2.11)	1.32(0.70–2.51)
**Education**				
None/Primary	42 (36.2)	74 (63.8)	1.0	--
Secondary	161 (42.0)	222 (58.0)	1.16 (0.89–1.52)	--
Tertiary	68 (35.1)	126 (65.0)	0.97 (0.71–1.32)	--
**Primary drug**				
Alcohol	126 (33.4)	251 (66.6)	1.0	1.0
Cannabis	49 (34.5)	93 (65.5)	1.03 (0.79–1.35)	1.14 (0.81–1.60)
Cocaine	47 (44.8)	58 (55.2)	1.34 (1.04–1.73)	0.99 (0.68–1.46)
Opioids	49 (62.0)	30 (38.0)	1.86 (1.48–2.32)	1.43 (1.04–1.96)*
Other	12 (34.3)	23 (65.7)	1.03 (0.63–1.66)	1.31 (0.82–2.08)
**Religion**				
Catholic	92 (37.4)	154 (62.6)	1.0	--
Protestant	127 (39.1)	198 (60.9)	1.04(0.84–1.29)	--
Muslim	46 (46.0)	54 (54.0)	1.23 (0.94–1.61)	--
Other	16 (30.8)	36 (69.2)	0.82 (0.53–1.28)	--
**Marital status**				
Single	105 (36.1)	186 (63.9)	1.0	--
Married	39 (31.0)	87 (69.1)	0.86 (0.63–1.16)	--
Other	43 (48.9)	45 (51.1)	1.35 (1.04–1.76)*	--
**Type of facility**				
Public-Butabika Hosp.	114 (30.0)	266 (70.0)	1.0	1.0
Private/NGO	169 (46.7)	193 (53.3)	1.56 (1.29–1.88)***	1.38 (1.05–1.82)*
**HIV status** †				
Positive	37 (55.2)	30 (44.8)	1.0	1.0
Negative	232(40.6)	340 (59.4)	0.73 (0.58–0.93)*	0.82 (0.61–1.10)

† HIV results were available for only 657 clients.

PR=Prevalence ratio

### Source of payment for previous treatment (Rehabilitation of alcohol/drug abuse)

**Figure 7:**
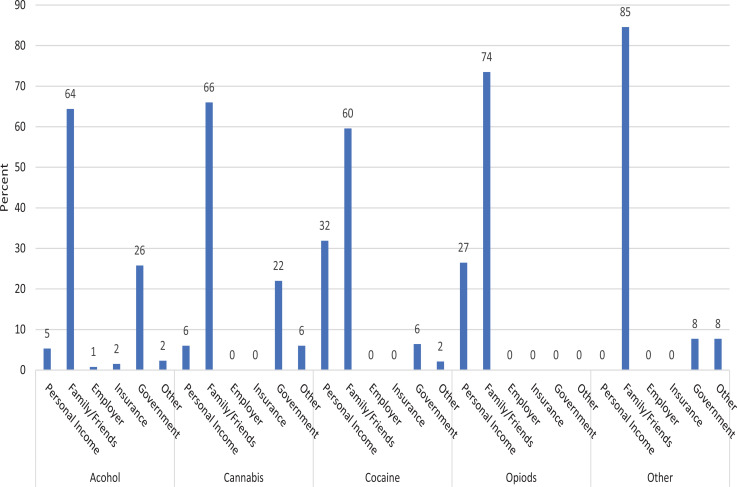
Source of payment for treatment by different kind of substance used

Overall, the main source of funds paid for treatment in the rehabilitation centres was family/friends. Of the 291 that had been treated for AOD before 193(66%) had their bills cleared by family and friends. Other sources of funds that paid for previous treatment were government (17%) and personal savings 13%. A closer examination of source of payment for previous treatment by the prime substance used shows that family and friends were a dominant source of funding (Figure 7). Government paid for 26% and 22% of the clients with alcohol and cannabis addictions respectively. Personal savings were used to pay for a third (32%) and 27% of the clients with cocaine and opioids addictions respectively.

### Expenditure on drugs and alcohol per week

Majority of clients (72%) spent between Shs 10,000 (USD 2.70) to 100,000 (USD 27) on alcohol or other drugs per week and this money was mainly either from own savings (86%) or family/friends (38%).

### Impact of substance use on clients and their families

The most commonly reported negative effects of substance use were mental health complications (40%), being arrested (30%), losing a job (29%) and developing physical complications (28%).

At family level, the most commonly reported negative effects of substance use were lack of basic needs (40%), spousal separation (15%) and domestic violence (35%). Strength and limitations of the study

The strength of the paper lies in the content, recency, focus on clients of rehabilitation centres, the length of data collection and data analysis. The content of the data is rich with information that includes characterization of the clients, AOD practices, kinds of drugs taken, expenditure, referrals, trend over time and analysis of treat treatment. The data are most recent and are collected in the time of the COVID-19 world epidemic. The focus on clients of the rehabilitation centres is new. The authors are not aware of any similar work that has been done before. The data were collected over a period of 8 months and this allows some assessment of temporal changes. The limitations of the study lie in the kind of clients in then centres and the timing of the study. The people that seek care in rehabilitation centres may not represent the general population of people with addiction problems. These can pay the daily fees of the private centres which many will not afford. The only public facility in the study offers free services but there are costs of maintaining attendants of the patient and purchase of commodities that the government cannot provide. The 8-month duration of data collection is not long enough to allow analysis of full seasonality and trend analysis. Then, being a COVID-19 time, one is not sure whether what we observe in the data wouldn't have been different without COVID-19.

## Discussion

The results show that clients of the rehabilitation centres in Kampala metropolitan area are largely youth, male, single, unemployed, urban and have attained secondary. There was a decline in number of clients from the month of November to December, 2019. The number of clients then rose to peak in February, 2020 but reduced with onset of the COVID-19 pandemic, which later rose again after two months. The most commonly used drugs were alcohol, cannabis and cocaine and clients took them almost daily. The commonest source of drugs were street dealers and friends. The overall age at first use of primary drugs was 20 and it was lower among men and those who took cannabis. Peer pressure was a major reason for start and continued use of drugs. Other reason for continued use is a need for leisure or feeling better. Injecting drugs users were 9% and a third of them shared equipment. Relapse rate is high as 38% were on repeat treatment while 23% had been treated 3 or more times before. Clients receive medical, psychological, prayer, traditional kinds of treatment. Major sources of funds for treatment were family/friends and personal savings.

The characteristics of the clients of the rehabilitation centers appear similar to those in previous studies with a few variations. For example, one similar study in Pakistan found a high percent of illiterate clients in rehabilitation centres^16^ while in this study majrity are educated.

Reduction of clients in December may be attributed to Christmas holidays when workers in the rehabilitation centres break off and thus only a few can be admitted. The peak in February may be explained by lots of alcohol and drug intake during the Christmas and New Year festivities that can lead to relapse among those with history of drug abuse.

Relatively high level of use of cocaine and opioids by women need further investigation. However, a systematic review study found that ratio of cannabis use prevalence between males and females has decreased significantly over time^17^.

The type of drugs used in urban areas in Uganda such as cannabis, cocaine and opioids have been reported in several studies^18^. It's surprising that the pattern of primary drugs used among clients of rehabilitation centres in the country (52% for alcohol, 19% cannabis and 14% cocaine) is nearly similar to that in South Africa (51% for alcohol, 21% cannabis and 9.6% cocaine. This paper adds details about changes over time and frequency of use of the drugs on existing amount of knowledge. Higher frequency of use of alcohol, cocaine compared to other drugs may be connected to availability and lower cost.

Lower age at first use of drugs among cocaine users compared to users of other drugs needs further investigation. However, the mean age is not very different from studies in Australia (17.6 years)^19^.

Prevalence of injecting drug use (IDU) (9%) among clients of rehabilitation centres adds on new knowledge about drug use. Several studies have reported dearth of evidence on IDU in the country^20^. The national population level of injecting drug use is estimated at 10 per 100,000 inhabitants^21^.

The proportion of those on repeat treatment at 38% appear quite high if we use it as an indicator of relapse among the clients. A study carried out earlier in Butabika national mental health hospital showed a relapse of 25%^22^. The associations of being on repeat treatment and being a man, taking Opioids, working as casual labourer/self-employed and seeking care in private facilities are not common in several studies. A study in 2010 in India found repeat treatment associated with unemployment, ever been marriage, higher age group^23^. A study published in 2013 in Pakistan showed that relapse is strongly associated with being female^24^. On preference of private facilities to the only public facility there is a lot of stigma at the later because it's the only national mental health referral hospital and has long been known as a place for “mad” people^25^. All the private facilities are much younger than the public facility and most of them are not even known

## Conclusions

Clients in these facilities are mostly young, male, single, urban and educated. The main substance of abuse is alcohol followed by cannabis and cocaine. Families/friends are a commonest source of funds that pay for rehabilitation services. The drugs are commonly used daily and this is instigated largely by peer pressure, leisure as well as a need to feel better. Street dealers are the major source of drugs while sharing of equipment among injecting drug users was high. Repeat treatment was associated with being male, seeking care in private facilities, being casual labourer/self-employed. The COVID-19 lockdown time covered in this study did not significantiy change the rate of consumption of AOD with exception of legal opiods. There is minimal access to Hepatitis C testing services.

## Recommendations

From the results in the study, we recommend the following: There is a need by the police and other relevant institutions to identify hotspots where the drugs are sold and design interventions to minimise or eliminate sales on these places. The ministry of health (MOH) should avail Hepatitis C testing services to rehabilitation centres. The program initiatives against substance use should target street drug dealers and friends of the AOD users. MOH and other players should provide target interventions for the male educated youth especially supporting their fight against peer pressure and unemployment. There is a need to identify and study those people in the communities who have AOD use disorders but don't go to the rehabilitation facilities. Information about them can be compared with that from the facilities and this will complete the whole picture of AOD abuse in the communities. One possible reason for failure to go the facilities is lack of funds and this could be factored in a probably case control study that can compare the two groups. Another area of future research can focus on why repeat treatment was associated with being male, seeking care in private facilities and being casual labourer/self-employed
